# Evaluation of genetic diversity among strains of the human gut commensal *Bifidobacterium adolescentis*

**DOI:** 10.1038/srep23971

**Published:** 2016-04-01

**Authors:** Sabrina Duranti, Christian Milani, Gabriele Andrea Lugli, Leonardo Mancabelli, Francesca Turroni, Chiara Ferrario, Marta Mangifesta, Alice Viappiani, Borja Sánchez, Abelardo Margolles, Douwe van Sinderen, Marco Ventura

**Affiliations:** 1 Laboratory of Probiogenomics, Department of Life Sciences, University of Parma, Italy; 2 GenProbio srl, Parma, Italy; 3 Departamento de Microbiologia y Bioquimica de Productos Lacteos, IPLA – CSIC, Villaviciosa, Asturias, Spain; 4 APC Microbiome Institute and School of Microbiology, National University of Ireland, Cork, Ireland

## Abstract

Bifidobacteria are members of the human gut microbiota, being numerically dominant in the colon of infants, while also being prevalent in the large intestine of adults. In this study, we determined and analyzed the pan-genome of *Bifidobacterium adolescentis*, which is one of many bacteria found in the human adult gut microbiota. *In silico* analysis of the genome sequences of eighteen *B. adolescentis* strains isolated from various environments, such as human milk, human feces and bovine rumen, revealed a high level of genetic variability, resulting in an open pan-genome. Compared to other bifidobacterial taxa such as *Bifidobacterium bifidum* and *Bifidobacterium breve*, the more extensive *B. adolescentis* pan-genome supports the hypothesis that the genetic arsenal of this taxon expanded so as to become more adaptable to the variable and changing ecological niche of the gut. These increased genetic capabilities are particularly evident for genes required for dietary glycan-breakdown.

Bifidobacteria are a common component of the microbiota of the human gastrointestinal tract (GIT) and, in particular, they are amongst the first bacterial colonizers of the intestine of neonates^1^,^2^,^3^. Bifidobacteria represent Gram positive, non-motile and non-spore-forming bacteria that belong to the phylum Actinobacteria^4^. Currently, genome sequences of one or more representatives of all 48 bifidobacterial species are available, which revealed a common saccharolytic genotype that is centred around a shared fermentative metabolic pathway particular to the *Bifidobacterium* genus and for this reason designated the bifid shunt. Furthermore, genome-based analyses revealed that bifidobacteria follow varying genetic strategies to adapt to their particular ecological niche(s), many of which relate to the mammalian GIT^5^,^6^. Among the bifidobacteria that colonize the human gut, strains of *Bifidobacterium adolescentis* appear to specifically colonize the gut of adult individuals and for this reason they represent a key bifidobacterial taxa of adult-associated bifidobacteria^7^. Recently, preliminary genetic and phenotypic characterization of the *B. adolescentis* species has been carried out, revealing their extensive capabilities to metabolize diet-derived glycans, in particular starch and starch-related/derived poly- and oligo-saccharides, such as amylopectin, pullulan, maltotriose and maltodextrin^8^.

Pan-genomes of two other human gut bifidobacterial species, *Bifidobacterium bifidum*^9^ and *Bifidobacterium breve*^10^, have previously been characterized. Notably, these analyses showed a closed pan-genome structure for both of these two species, revealing the presence of specific genetic strategies to establish and persist in the human gut, such as through the production of various types of pili^11^,^12^,^13^,^14^ or metabolic capabilities toward particular host-glycans^9^,^15^,^16^.

In contrast, the current genomic information available for *B. adolescentis* is rather limited, being represented by six genome sequences, of which three that are still fragmented in many contigs^6^,^8^,^17^. Thus, the genetic knowledge and understanding of the metabolic capabilities of this taxon is still in its infancy. In this study, we report on the genome sequences of twelve *B. adolescentis* strains that had been isolated from the adult gut or from rumen. Comparative genomic analyses of these sequences together with six other publicly available genome sequences of *B. adolescentis* species was performed. In addition, carbohydrate profiling of these strains was achieved involving various glycans including dietary- as well as host-derived glycans. Dietary changes are expected to impact on the ecological properties of the mammalian gut and thus on microbiota composition. The *B. adolescentis* taxon was shown to exhibit, through *in silico* and *in vitro* experiments, more extensive genetic flexibility and potential adaptive competitiveness to this highly variable ecological niche compared to other human bifidobacterial species.

## Results

### General genome features of *B. adolescentis* species

In order to evaluate the genetic content of the *B. adolescentis* species, we isolated eight strains in addition to four strains (obtained from international collections) belonging to this taxon from different ecological niches, including human feces, human milk, and bovine rumen (Table 1). The genome sequences of these strains were decoded through a Next Generation Sequencing (NGS) approach and subjected to comparative genomic analyses together with five other publicly available *B. adolescentis* genomes, corresponding to strains ATCC15703, 22L, BBMN23, 150 and L2-32. In addition, we included the chromosome sequence of *B. stercoris* JCM15918^5^, since the latter microorganism has recently been re-classified as *B. adolescentis* JCM15918^18^. These twelve genome sequences of newly isolated and previously acquired *B. adolescentis* strains were sequenced to a coverage depth that ranged from 43.6-fold to 289.9-fold, which upon assembly resulted in thirty three to seven contigs, respectively (Table 1). Using the genome of the *B. adolescentis* type-strain ATCC15703 as a reference sequence, we were able to determine the presumed contig orientation and order for each genome draft. As outlined in Table 1, the number of predicted ORFs in each genome ranged from 1614 for *B. adolescentis* LMG11579 to 2215 for *B. adolescentis* 487B. In contrast to what has been observed for other bifidobacterial taxa for which three or more genomes are available, such as *B. bifidum*^9^, *B. breve*^10^, *B. longum* subsp. *longum*^19^ and *Bifidobacterium animalis* subsp. *lactis*^20^, the differences among the ORFomes from different *B. adolescentis* members were shown to be higher, suggesting that this bifidobacterial taxon exhibits a more extensive level of genetic diversity compared to that observed for other currently available bifidobacterial pan-genomes.

Notably, functional classification of the *B. adolescentis* ORFome based on the eggNOG database^21^ was possible for 89.3% of the predicted ORFs. No function could be assigned to the remaining 10.7% and these identified ORFs were therefore annotated as hypothetical proteins. Furthermore, the eggNOG classification of the *B. adolescentis* genomes revealed that the majority of genes for which a function could be assigned are predicted to be involved in housekeeping functions, amino acid and carbohydrate metabolism, and associated transport activities (Fig. S1), similar to what had been observed for other members of the *Bifidobacterium* genus^5^,^6^,^22^. Our findings furthermore highlight that, being consistent with observations for other (sub)species of the genus *Bifidobacterium*, chromosomal sequences of the *B. adolescentis* species support the view that also this bifidobacterial taxon has adopted a saccharolytic life style.

### Phylogenomic analyses of *B. adolescentis* species

**Figure 1:**
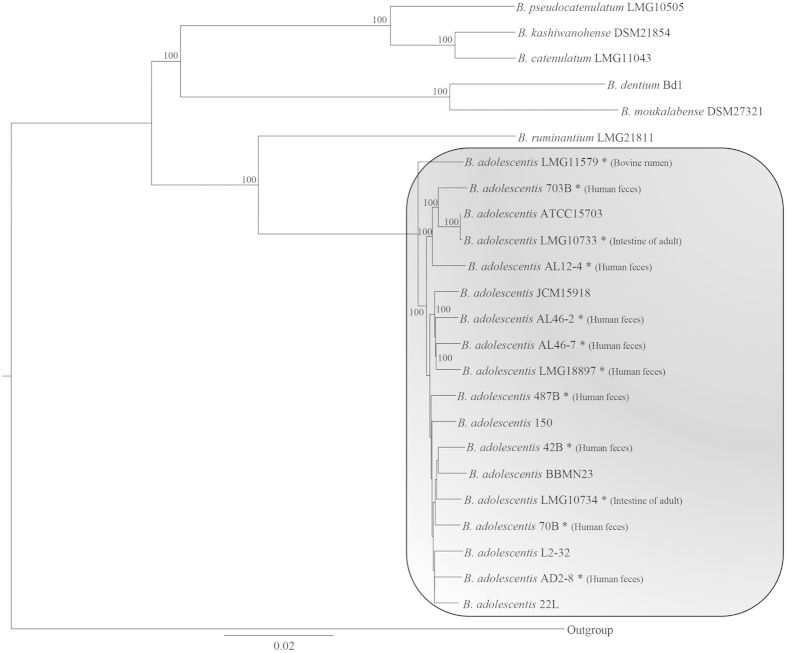
Genetic relationship within the *B. adolescentis* group. The phylogenetic supertree was generated based on sequence similarities among 872 orthologous genes that are present in all analyzed strains.

The availability of the genome sequences of the type strains from all (currently described) 48 (sub)species of the *Bifidobacterium* genus allows a robust reconstruction of the phylogeny among members of this genus^6^. Thus, a comparative analysis was undertaken to identify orthologous genes among the bifidobacterial genome sequences of the strains belonging to the *B. adolescentis* species, as well as among the strains belonging to the *B. adolescentis* group^2^, which include *Bifidobacterium dentium*, *Bifidobacterium pseudocatenulatum, Bifidobacterium catenulatum, Bifidobacterium moukalabense, Bifidobacterium kashiwanohense, Bifidobacterium ruminantium* and *B. stercoris/adolescentis*. These analyses revealed the existence of 872 orthologous genes, which are shared among sequenced members of the *B. adolescentis* group. A concatenated protein sequence deduced from these orthologous genes of the *B. adolescentis* group was constructed in order to build a *B. adolescentis* group supertree (Fig. 1), which demonstrated that all 18 *B. adolescentis* strains are positioned on sub-branches of the same cluster. Interestingly, the *B. adolescentis* cluster includes a separate branch encompassing the genome of *B. adolescentis* LMG11579, which was isolated from a bovine rumen. This branch appears to be phylogenetically distinct from the other *B. adolescentis* strains that were isolated from various human samples, thus suggesting that *B. adolescentis* strain differences reflect the ecological origin of such investigated strains.

### Pan-genome and core-genome analysis of *B. adolescentis* species

**Figure 2:**
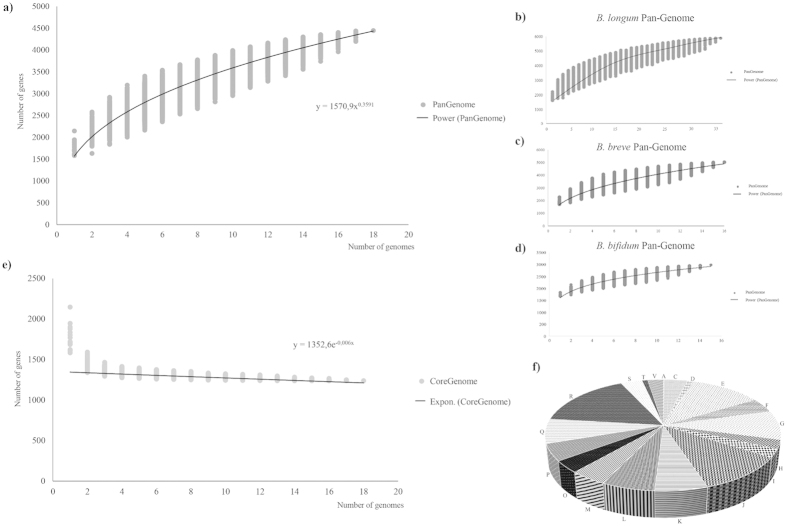
Pan-genome and core-genome of the *B*. *adolescentis* species. The pan-genome of *B. adolescentis* species (panel **a**) as well as *B. longum, B. breve* and *B. bifidum* species (panel **b**–**d**) and core-genome of *B. adolescentis* species (panel **e**) are represented as variation of their gene pool sizes upon sequential addition of genomes analysed. Panel (**f**) shows functional assignment of the *B. adolescentis* core genome based on the eggNOG database. Each letter stands for the following function: **J**, Translation, ribosomal structure and biogenesis, **A**, RNA processing and modification, **K**, Transcription, **L**, Replication, recombination and repair, **D**, Cell cycle control, cell division, chromosome partitioning, **V**, Defense mechanism, **T**, Signal transduction mechanisms, **M**, Cell wall/membrane/envelope biogenesis, **O**, Posttranslational modification, protein turnover, chaperones, **C**, Energy production and conversion, **G**, Carbohydrate transport and metabolism, **E**, Amino acid transport and metabolism, **F**, Nucleotide transport and metabolism, **H**, Coenzyme transport and metabolism, **I**, Lipid transport and metabolism, **P**, Inorganic ion transport and metabolism, **Q**, Secondary metabolites biosynthesis, transport and catabolism, **R**, General function prediction only, **S**, Function unknown.

The genome sequences of the 18 *B. adolescentis* strains were used to calculate the total gene repertoire encountered in this species. Thus, we evaluated the pan-genome and core-genome of the *B. adolescentis* taxon based on the clusters of orthologous genes (COGs) following a previously described method^23^. We identified a *B. adolescentis* pan-genome that consists of 4448 COGs (Fig. 2a). The pan-genome size, when plotted on a log-log scale as a function of the number of analyzed genomes, shows that the power trend line has not reached a plateau. Indeed, the number of genes discovered by sequential addition of genome sequences was reduced from 334-230 COGs in the first three genome additions, to 97-90 COGs in the final three additions. These findings indicate the occurrence of an open pan-genome within the *B. adolescentis* species, and suggest that full knowledge on the genetic diversity of this species has not yet been obtained^23^. This is in contrast with the determined pan-genomes of other human isolated bifidobacterial species, such as *B. bifidum*^9^, *B. breve*^10^ or *B. longum* subsp. *longum*^19^, which all displayed a closed structure (Fig. 2b–d). Moreover, the genome sequences of other bifidobacterial species showed that the ORF numbers of *B. bifidum* and *B. breve* genomes range from 1689 to 1835 and from 1748 to 1915, respectively^10^,^24^. In contrast, among representatives of the *B. adolescentis* species the size differences between individual ORFomes are larger, ranging from 1614 to 2215 (Table 1). The functional classification of the genes that do not belong to the *B. adolescentis* core-genome revealed that a large part of these genes have an unknown function, followed by genes involved in transport and metabolism of carbohydrates (Fig. S2). This indicates that genomes of the *B. adolescentis* species possess a higher level of genetic diversity as compared to those of other human-derived bifidobacteria. Furthermore, analysis of the set of predicted COGs allowed the identification of 1238 genes shared by all analysed *B. adolescentis* genomes, representing the core-genome of the *B. adolescentis* species (Fig. 2e). Examination of functional classification distribution among this core-genome, based on the eggNOG database^21^, suggests that a large proportion of these identified core genes are associated with housekeeping functions, and amino acid and carbohydrate metabolism, and corresponding transport (Fig. 2f).

### Core-genome sequences of *B. adolescentis* species

**Figure 3:**
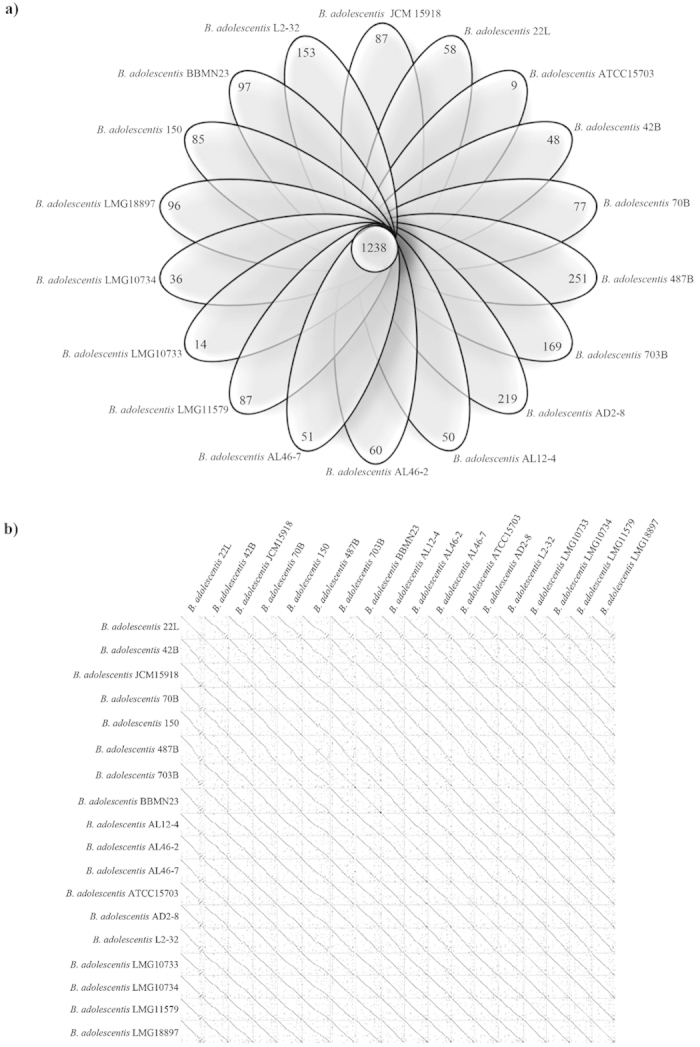
Comparative analysis of *B. adolescentis* genomes. Panel (**a**) displays the Venn diagram representing the unique and orthologues genes between the 18 *B. adolescentis* genomes. Panel (**b**) shows the synteny plot alignment between the 18 *B. adolescentis* genomes.

Comparative genome analyses based on the 18 *B. adolescentis* genomes revealed the occurrence of shared orthologous genes as well as unique genes. As described above and employing the criteria outlined in the Materials and Methods, *in silico* analyses identified that 1238 genes are common among these strains, of which 369 were also common among other (sub)species of the genus *Bifidobacterium*. Furthermore, a varying number of unique genes, ranging from nine for *B. adolescentis* ATCC15703 to 251 for *B. adolescentis* 487B, were detected (Fig. 3a), representing the presumed Truly Unique Genes (TUG) of each *B. adolescentis* strain. Moreover, *in silico* analysis of the functional classification of TUGs failed to identify any correspondence between the predicted function of TUGs and the particular ecological origin of the *B. adolescentis* strain in question. Using an *in silico* approach to predict average nucleotide identity (ANI) values between *B. adolescentis* genomes^25^, we showed a highly syntenic genome structure among members of this species, with associated ANI values ranging from 97.77% to 99.93%, validating that *B. adolescentis* genomes belong to the same species^6^ (Table S1). Furthermore, this highly syntenic structure of *B.adolescentis* genome sequences was confirmed by comparative analyses using Dot-plot alignments (Fig. 3b). These analyses showed an apparent genome inversion in the chromosome of *B. adolescentis* 22L that was validated by PCR (data not shown). Furthermore, the analysis of this region revealed the presence of TUGs that appeared to have been acquired by horizontal gene transfer (HGT; see also below) as well as genes that are included in the core genome. These genes encode hypothetical proteins, putative phage proteins, several carbohydrate transport systems, enzymes that are involved in carbohydrate metabolism and the F1F0-ATPase cluster.

Finally, the analysis of the core genome sequences of the *B. adolescentis* taxon allowed the identification of three core genes that are uniquely present in this species (i.e. absent in any of the other analysed bifidobacterial genomes), thus representing unique *B. adolescentis* core genes and encoding a Major Facilitator Superfamily (MFS) transporter, an acyltransferase and a hypothetical protein.

### Mobilome of the chromosomes of *B. adolescentis* species

The mobilome represents the total number of genes that may have been acquired by HGT and its identification was performed using the software suite COLOMBO v3.8 implemented by SIGI-HMM^26^ and DarkHorse software^27^. The obtained results were merged, revealing that 13% of all ORFs were predicted to represent HGT-acquired genes. Furthermore, the observed percentage of ORFs putatively acquired by HGT events and predicted to be TUGs ranged from 3.09% in the genome of *B. adolescentis* ATCC15703 to over 40% in the chromosomes of *B. adolescentis* 70B and *B. adolescentis* AD2-8, respectively (Table S1). The predicted mobilome of *B. adolescentis* highlights a rich arsenal of insertion sequences (IS) and an abundance of prophage-like elements. Additional putative mobile elements identified in the *B. adolescentis* genomes are represented by CRISPR loci and a Restriction Modification (R/M) system. Other variable regions were shown to include an EPS gene cluster and finally a type IVa pilus biosynthesis gene cluster (Fig. S2). The type IVa pilus biosynthesis gene cluster was previously described in *B. adolescentis* 22L^8^ and its presence is also identified in *B. adolescentis* LMG10734 (locus tags LMG10734_1251-LMG10734_1263), *B. adolescentis* LMG18897 (locus tags LMG18897_1585-LMG18897_1597), *B. adolescentis* 15O (locus tags 15O_1475-15O_1485), *B. adolescentis* 42B (locus tags 42B_1313-42B_1323), *B. adolescentis* 70B (locus tags 70B_1301-70B_1311), *B. adolescentis* 487B (locus tags 487_1645-487_1655), *B. adolescentis* AD2-8 (locus tags AD28_1414-AD28_1424) and *B. adolescentis* AL46-2 (locus tags AL46-2_1300-AL46-2_1310). The function of this cluster is unknown, yet in other microbes it has been linked to motility, conjugation, adherence and DNA uptake^28^. Overall, these findings suggest that the genetic diversity found in *B. adolescentis* species is driven by various processes including conjugation, transformation, as well as phage-mediated transduction, which is consistent with previous publications^29^,^30^,^31^,^32^,^33^.

### The glycobiome of *B. adolescentis* species

**Figure 4:**
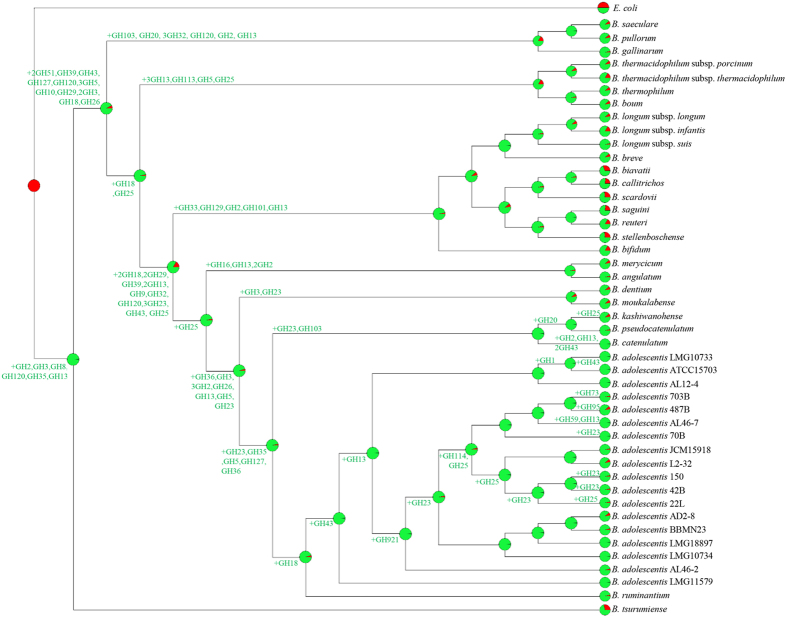
Reconstruction of gene gain and loss events among the analyzed *B. adolescentis* strains. A tree was constructed using information related to the presence or absence of COGs for the whole *B. adolescentis* pan-genome. Each node is marked by a pie diagram showing the acquired COGs (in red) and the COGs derived from the previous node (in green). Furthermore, the number of members of GH families that had been acquired is indicated next to each diagram.

**Figure 5:**
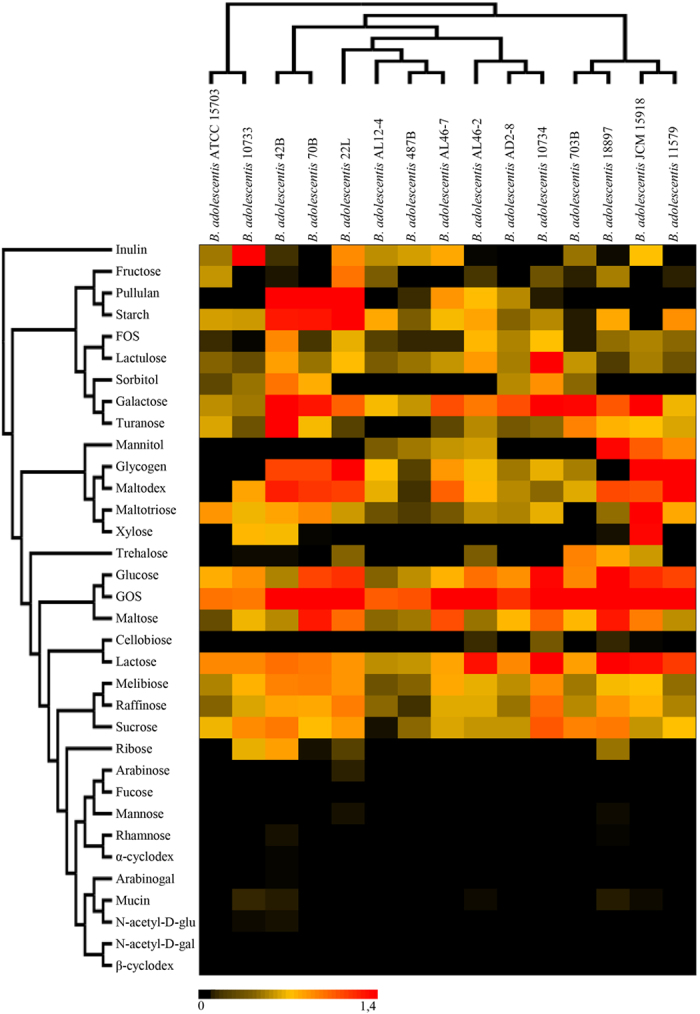
Evaluation of carbohydrate utilization by *B. adolescentis* strains. The heat map shows the growth performance of *B. adolescentis* strains on different carbon sources. Cultures were grown in biologically independent triplicates. The different shading represents the optical density reached by the various culture.

Thanks to their saccharolytic enzyme arsenal bifidobacteria are in a position to metabolize a wide range of carbohydrates (as a carbon and energy source), ranging from dietary- as well as host-derived glycans^8^,^22^,^34^,^35^. In order to extend our knowledge on the carbohydrate fermentation capabilities of *B. adolescentis* species, we performed an *in silico* prediction, in accordance to Carbohydrate-Active enZYmes Database (CAZy) database^36^, involving all 18 sequenced *B. adolescentis* genomes. This analysis revealed that the pan-genome of *B. adolescentis* contains genes encoding predicted carbohydrate-active enzymes, including 36 glycosyl hydrolase (GH) families, 12 glycosyl transferase (GT) families and four carbohydrate esterase (CE) families (Fig. S3). Interestingly, all 18 analyzed *B. adolescentis* genomes encompass genes predicted to encode enzymes involved in the uptake and utilization of plant-derived carbohydrates such as starch and starch-like oligo/polysaccharides, like maltodextrin, maltotriose, amylose, glycogen and pullulan^37^. These enzymes were previously identified to represent a distinctive characteristic of *B. adolescentis* 22L genome, supporting a superior growth performance of this strain compared to most other bifidobacteria when cultivated on starch or starch-like oligo/polysaccharides^8^. Consistent with this work, our analysis revealed a high abundance of genes belonging to the GH13 family (ranging from 14 in *B. adolescentis* 70B, *B. adolescentis* 703B, *B. adolescentis* ATCC15703 and *B. adolescentis* LMG10733, to 18 in *B. adolescentis* LMG10734 and *B. adolescentis* AL46-2), which encompasses enzymes with predicted α-glucosidase, amylase, pullulanase, and cyclomaltodextrinase activities. *In silico* analyses also highlighted the presence of a number of GHs (e.g., GH10, GH29, and GH59) that appear to be present in just a single strain of *B. adolescentis* species (Fig. S3a), and their annotation suggests that they may encode hydrolytic activities and thus carbohydrate-related metabolic abilities that are unique to such strains. We have further predicted the evolution of the GH arsenal of *B. adolescentis* pan-genome using BlastGraph^38^. This analysis allows the generation of a tree based on information regarding the presence or absence of COG families in each of the bifidobacterial strains assayed through the use of the dollo-parsimony algorithm^39^. Notably, this analysis predicted the early acquisition of GH43 and GH13 family members during the evolution of the *B. adolescentis* species (Fig. 4), which are crucial for the degradation, respectively, of plant polysaccharides^36^, and glycans with α-glucosidic linkages such as plant-derived starch or glycogen. Interestingly, *B. adolescentis* LMG11579, which is the only *B. adolescentis* strain so far isolated from bovine rumen, was predicted to neither have gained nor lost members of this GH13 COG. Furthermore, exploration of the functional evolution of this species showed widespread acquisition of COGs classified as GH23 and GH25 at multiple nodes. These GH families encode lysozymes that are often carried by phage genomes and expressed during the lytic phase, thus their acquisition during the evolution of *B. adolescentis* species may be linked to frequent interactions with phages populations (http://www.cazy.org/Glycoside-Hydrolases.html). In order to validate the above mentioned genomic-based analyses, we carried out growth experiments of *B. adolescentis* strains on 33 carbohydrates including both plant- and host-derived glycans, as unique carbon sources (Fig. 5). As displayed in Fig. 5 all tested strains are able to ferment a common set of sugars, such as galactose, glucose, galactooligosaccharides (GOS), lactose, lactulose, maltodextrin, maltose, maltotriose, melibiose, and raffinose. In contrast, fermentation capabilities for other sugars including fructooligosaccharides (FOS), fructose, glycogen, mannitol, pullulan, ribose, sorbitol, trehalose, turanose and xylose were shown to be variable among the strains tested. Furthermore, in contrast to other *B. adolescentis* strains, *B. adolescentis* 703B and *B. adolescentis* JCM15918 strains did not exhibit any appreciable growth on starch (Fig. 5).

Notably, none of the *B. adolescentis* strains assayed here was shown to be capable of utilizing mucin, N-acetyl-D-galactosamine, N-acetyl-D-glucosamine or fucose, which indicates that the tested *B. adolescentis* strains possess limited metabolic capabilities to with regards to (monosaccharide components of) host-derived glycans (Fig. 5). Such metabolic features are commonly found among bifidobacterial taxa associated with the early stages of life in mammals such as *B. bifidum*, *B. longum* subsp. *infantis* and *B. breve*^9^,^15^,^16^,^40^,^41^. It is not unexpected that these metabolic characteristics are absent and/or have been lost by a bifidobacterial species that is typically found in the adult-colon such as *B. adolescentis*. This is also further supported by the absence in the *B. adolescentis* pan-genome of genes encoding sialidases, sialic acid metabolism, fucosidases, the relative paucity of beta-galactosidase-encoding genes and the absence of homologues of the LNB/GNB utilization cluster. This suggests that this species has specialized itself towards the utilization of plant-derived glycans, in particular (resistant) starch and starch-like polysaccharides, that are normally present in high abundance in the diet of adults.

## Discussion

The increasing number of publicly available bifidobacterial genomes offers the possibility to understand the evolution and the genomic diversity within this genus^5^,^6^. In this study, we analyzed the genomic diversity of 12 different *B. adolescentis* strains combined with six additional, publicly available *B. adolescentis* genomes. These results allowed the identification of an open pan-genome of the *B. adolescentis* species, which is in contrast to what has been observed for other human gut bifidobacterial species such as *B. bifidum*, *B. breve*, *B. longum* subsp. *longum* and *B. animalis* subsp. *lactis*^9^,^10^,^19^,^20^. This indicates that the *B. adolescentis* taxon exhibits greater genetic diversity compared to other human gut bifidobacterial (sub)species. Furthermore, the pan-genome of the *B. adolescentis* species is enriched in genes that are predicted to be involved in the metabolism of dietary, plant-derived glycans, in particular starch and starch-like oligo- and poly-saccharides, except in the case of two strains, *B. adolescentis* 703B and *B. adolescentis* JCM15918. In contrast, the *B. adolescentis* genomes do not appear to encode genes involved in the metabolism of host-derived glycans such as mucin and human milk oligosaccharides. These results supports the notion that the (human-associated) *B. adolescentis* species has evolved towards an ecological niche where plant-polysaccharides are present in high abundance, such as the large intestine of adult human beings. However, caution should be taken when drawing inferences based *in silico* predictions and *in vitro* experiments. Thus, future *in vivo* analyses performed in murine models or clinical trials will be needed in order to validate the results described in this study.

## Methods

### Bacterial strains, growth conditions and chromosomal DNA extraction

The strains used in this study are listed in Table 1. *B. adolescentis* cultures were incubated in an anaerobic atmosphere [2.99% (vol/vol) H_2_, 17.01% (vol/vol) CO_2_, and 80% (vol/vol) N_2_] in a chamber (Concept 400, Ruskin) in de Man-Rogosa-Sharpe (MRS) (Scharlau Chemie) supplemented with 0.05% (wt/vol) L-cysteine hydrochloride and incubated at 37 °C for 16 h. Bacterial DNA was extracted as described previously^42^ and subjected to further phenol/chloroform purification using a previously described protocol^43^.

### Genome sequencing and data assembly and bioinformatics analyses

All genomes used for this study were determined by GenProbio srl (Parma, Italy) using the MiSeq Illumina (Illumina, USA). Genomic libraries were constructed employing the TruSeq DNA PCR-Free LT Kit (Illumina) and using 2.5 μg of genomic DNA, which was fragmented with a Bioruptor NGS ultrasonicator (Diagenode, USA) followed by size evaluation using Tape Station 2200 (Agilent Technologies). Library samples were loaded into a Flow Cell V3 600 cycles (Illumina) according to the technical support guide, and generated reads were depleted of adapter sequences, quality filtered and assembled through the MEGAnnotator pipeline^44^. The overall generated sequencing output per genome ranged from 78 Mb to 515 Mb, approximately corresponding to a 43.6- to 289.9-fold genomic coverage, respectively.

Protein-encoding open reading frames (ORFs) were predicted using a combination of the methods Prodigal^45^ and BLASTX^46^. Results of the two gene finder programs were then automatically annotated on the basis of BLASTP^47^ analysis using *B. adolescentis* ATCC15703 as the reference genome (NCBI Accession Number: AP009256). Functional assignment was performed and manually edited based on similarity searches against the non-redundant protein database provided by the National Centre for Biotechnology Information. Artemis^48^ was employed to inspect the results of the ORF finding efforts and associated BLASTP results, and used for manual editing in order to check, or if necessary, redefine the start of every predicted coding region, or to remove or add coding regions. Furthermore, the revised gene-protein set was searched against the Swiss-Prot (http://www.expasy.ch/sprot//TrEMBL), PRIAM (http://priam.prabi.fr/), protein family (Pfam; http://pfam.sanger.ac.uk/), TIGRFAMs (http://www.jcvi.org/cms/research/projects/tigrfams/overview/), Interpro (INTERPROSCAN; http://www.ebi.ac.uk/Tools/InterProScan/), Kyoto Encyclopedia of Genes and Genomes (KEGG; http://www.genome.jp/kegg/), and COG (http://www.ncbi.nlm.nih.gov/COG/) databases, in addition to BLASTP (http://blast.ncbi.nlm.nih.gov/Blast.cgi). Functional assignments were defined by manual processing of the combined results. Manual corrections to automated functional assignments were completed on an individual gene-by-gene basis as needed.

Ribosomal RNA genes were detected on the basis of BLASTN searches and annotated manually. Transfer RNA genes were identified using tRNAscan-SE^49^. Restriction/modification (R/M) systems were searched on the basis of the REBASE database^50^, Carbohydrate-active enzymes were identified based on similarity to the carbohydrate-active enzyme (CAZy) database entries^36^, transporter classification was performed according to the TC-DB scheme^51^ and Enzyme Commission (EC)/Gene Onthology (GO) annotation was assigned using annot8r^52^. Variances in GC content were profiled by the DNA segmentation algorithm hosted at http://tubic.tju.edu.cn/GC-Profile/53, atypical codon usage regions were mapped using the factorial correspondence analysis through the assistance of the GCUA software^54^.

### Comparative genomics

Each predicted proteome of a given *B. adolescentis* strain was searched for orthologues against the total proteome of *B. adolescentis* species, where orthology between two proteins was defined as the best bidirectional FASTA hits^55^. Identification of orthologues, paralogues, and unique genes was performed following a preliminary step involving the comparison of each protein against all other proteins using BLAST analysis^47^ (cutoff: E value of <1 × 10^−4^ and 30% identity over at least 80% of both protein sequences), and the resulting output was then clustered into protein families using MCL (graph theory-based Markov clustering algorithm)^56^. Following this approach, unique protein families encoded by the analyzed *B. adolescentis* genomes were classified. Protein families shared between all genomes, named core gene families, were defined by selecting the families that contained at least one single protein member for each genome.

Each set of orthologous proteins was aligned using CLUSTAL_W^57^, and phylogenetic trees were constructed using the maximum-likelihood in PhyML^58^. The supertree was built using FigTree (http://tree.bio.ed.ac.uk/software/figtree/).

### Pan-genome calculation

For all genomes used in this study, a pan-genome calculation was performed using PGAP^59^. The ORF content of each genome was organized in functional gene clusters using the gene family (GF) method. A pan-genome profile and a core genome profile were built using all possible BLAST combinations for each genome being sequentially added. Finally, using the pan-genome profile of shared orthologues between the *B. adolescentis* genomes, a pan-genome tree was constructed. This tree was visualized using FigTree (http://tree.bio.ed.ac.uk/software/figtree/).

### Prediction of gene acquisition and loss

Prediction and tree visualization of glycosyl hydrolase-encoding genes that had either been acquired or lost was performed with BlastGraph^38^. Data from BLASTP^46^ comparisons of all pan-genome-deduced proteins to each other were used as the input, where the clustering cut-off value was set at 50% identity over at least 50% of both protein sequences.

### Carbohydrate growth assays

Fifteen *B. adolescentis* strains were grown on semi-synthetic MRS medium supplemented with 0.5% (wt/vol) of a particular sugar and optical densities (OD at 600 nm) were recorded using a plate reader (BioTek, Winooski, VT, USA). The plate reader was run in discontinuous mode, with absorbance readings performed in 30 minutes intervals for 48 hours, where each reading was preceded by 30 s shaking at medium speed. Cultures were grown in biologically independent triplicates and the resulting growth data were expressed as the mean of these replicates. Carbohydrates were purchased from Sigma and Carbosynth (Berkshire, UK).

### Nucleotide sequence accession numbers

The sequences reported in this study have been deposited in the GenBank database (Accession Number SAMN04231308, SAMN04231309, SAMN04231310, SAMN04231311, SAMN04231312, SAMN04231313, SAMN04231314, SAMN04231315, SAMN04231316, SAMN04231317, SAMN04231318 and SAMN04231319).

## Additional Information

**How to cite this article**: Duranti, S. *et al.* Evaluation of genetic diversity among strains of the human gut commensal *Bifidobacterium adolescentis*. *Sci. Rep.* **6**, 23971; doi: 10.1038/srep23971 (2016).

## Supplementary Material

Supplementary Information

## Figures and Tables

**Table 1:** *Bifidobacterium adolescentis* strain list.

Strains	Source of isolation	Genome Size	No. of ORFs	Coverage	Contigs	Accession Number	Reference*
*B. adolescentis* 22L	Human milk	2203222	1725	/	/	NZ_CP007443.1	Duranti *et al.*^8^
*B. adolescentis* ATCC15703	Intestine of adult	2089645	1649	/	/	AP009256.1	NCBI database
*B. adolescentis* BBMN23	Human feces	2173720	1742	/	/	NZ_CP010437.1	NCBI database
*B. adolescentis* L2-32	Intestine of adult	2355465	1960	/	/	NZ_AAXD00000000.2	NCBI database
*B. adolescentis* 150	Human feces	2310538	1934	/	/	NZ_LBHQ00000000.1	Dyachkova *et al.*^17^
*B. adolescentis* LMG18897	Human feces	2146787	1751	289x	33	LNKM00000000	This study
*B. adolescentis* LMG11579	Bovine rumen	2061686	1614	290x	7	LNKL00000000	This study
*B. adolescentis* LMG10733	Intestine of adult	2084232	1654	139x	8	LNKJ00000000	This study
*B. adolescentis* LMG10734	Intestine of adult	2148629	1653	143x	20	LNKK00000000	This study
*B. adolescentis* 42B	Human feces	2212742	1855	120x	15	LNKB00000000	This study
*B. adolescentis* 70B	Human feces	2212129	1774	114x	14	LNKC00000000	This study
*B. adolescentis* 487B	Human feces	2597776	2215	76x	11	LNKD00000000	This study
*B. adolescentis* 703B	Human feces	2372808	2045	59x	33	LNKE00000000	This study
*B. adolescentis* AD2-8	Human feces	2379716	2029	44x	26	LNKF00000000	This study
*B. adolescentis* AL12-4	Human feces	2094907	1666	125x	8	LNKG00000000	This study
*B. adolescentis* AL46-2	Human feces	2208560	1770	115x	17	LNKH00000000	This study
*B. adolescentis* AL46-7	Human feces	2203026	1817	119x	12	LNKI00000000	This study
*B. stercoris/adolescentis* JCM15918	Adult feces	2304613	1890	/	/	JGZQ00000000	Lugli *et al.*^6^

^*^The references were based on the decoding genomes project according to NCBI database.
