# Supplementary material for: Evaluation of genetic diversity among strains of the human gut commensal Bifidobacterium adolescentis
Source: Sci Rep. 2016 Apr 1;6:23971. doi: 10.1038/srep23971 (PMC4817515; doi:10.1038/srep23971)
Supplement: Supplementary Information [file srep23971-s1.pdf]

Evaluation of genetic diversity among strains of the human gut commensal *Bifidobacterium adolescentis*

Sabrina Duranti[1], Christian Milani[1], Gabriele Andrea Lugli[1], Leonardo Mancabelli[1], Francesca Turroni[1], Chiara Ferrario[1], Marta Mangifesta[2], Alice Viappiani[2], Borja Sánchez[3¶], Abelardo Margolles[3], Douwe van Sinderen[4] and Marco Ventura[1]

Supplementary information

Fig. S1. Functional assignment of the analyzed *B. adolescentis* genomes based on eggNOG database. Each letter stands for the following function: **J**, Translation, ribosomal structure and biogenesis, **A**, RNA processing and modification, **K**, Transcription, **L**, Replication, recombination and repair, **D**, Cell cycle control, cell division, chromosome partitioning, **V**, Defense mechanism, **T**, Signal transduction mechanisms, **M**, Cell wall/membrane/envelope biogenesis, **O**, Posttranslational modification, protein turnover, chaperones, **C**, Energy production and conversion, **G**, Carbohydrate transport and metabolism, **E**, Amino acid transport and metabolism, **F**, Nucleotide transport and metabolism, **H**, Coenzyme transport and metabolism, **I**, Lipid transport and metabolism, **P**, Inorganic ion transport and metabolism, **Q**, Secondary metabolites biosynthesis, transport and catabolism, **R**, General function prediction only, **S**, Function unknown.

Fig. S2. Functional classification of the genes that do not belong to the *B. adolescentis* core-genome based on eggNOG database. Each letter stands for the following function: **J**, Translation, ribosomal structure and biogenesis, **A**, RNA processing and modification, **K**, Transcription, **L**, Replication, recombination and repair, **D**, Cell cycle control, cell division, chromosome partitioning, **V**, Defense mechanism, **T**, Signal transduction mechanisms, **M**, Cell wall/membrane/envelope biogenesis, **O**, Posttranslational modification, protein turnover, chaperones, **C**, Energy production and conversion, **G**, Carbohydrate transport and metabolism, **E**, Amino acid transport and metabolism, **F**, Nucleotide transport and metabolism, **H**, Coenzyme transport and metabolism, **I**, Lipid transport and metabolism, **P**, Inorganic ion transport and metabolism, **Q**, Secondary metabolites biosynthesis, transport and catabolism, **R**, General function prediction only, **S**, Function unknown.

Fig. S3. Mobile genetic elements of the analyzed *B. adolescentis* genomes. From inner to outer circle: the approximate genome sizes, percentage of genes acquired by horizontal gene transfer, number of unique genes, number of complete pilus type Iva loci, number of complete EPS cluster, number of R/M systems and number of CRISPR loci.

Fig. S4. Prediction of glycosyl hydrolases (GH), glycosyl transferases (GT) and carbohydrate esterases (CE) in *B. adolescentis* genomes. Panels a, b and c show, respectively, the GH and GT and

CE families profile of all analysed *B. adolescentis* genomes, based on the CAZy database. The color represents the numbers of genes identify for each GH, GT or CE families.

Table S1 Average nucleotide identity (ANI) alignment of *B. adolescentis* strain.

Table S2 Percentage of genes putatively involved in HGT events.

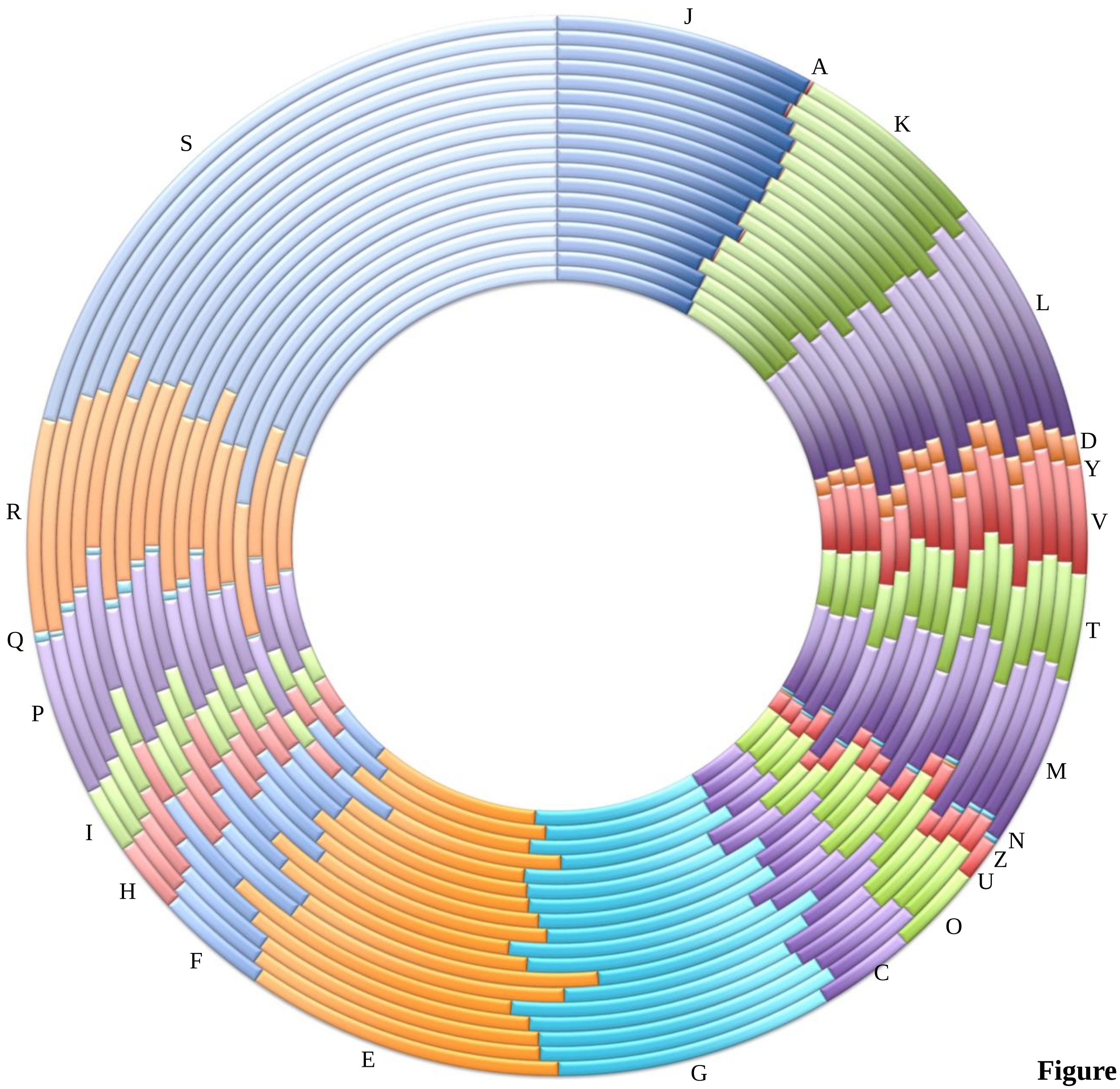

Figure S1

	B. adolescentis 150	*B. adolescentis* 22L	*B. adolescentis* 42B	*B. adolescentis* JCM15918	*B. adolescentis* 487B	*B. adolescentis* 703B	*B. adolescentis* 70B	*B. adolescentis* AD2-8	*B. adolescentis* AL12-4	*B. adolescentis* AL46-2	*B. adolescentis* AL46-7	*B. adolescentis* ATCC15703	*B. adolescentis* BBMN23	*B. adolescentis* L2-32	*B. adolescentis* LMG10733	*B. adolescentis* LMG10734	*B. adolescentis* LMG11579	*B. adolescentis* LMG18897
A				0.3%		0.3%			0.3%					0.2%				
C	1.7%	1.8%	2.4%	1.0%	1.1%	1.6%	2.2%	2.0%	1.7%	1.4%	1.0%	2.3%	1.7%	2.1%	3.0%	1.6%	1.3%	1.5%
D	1.2%	0.9%	0.5%	1.3%	3.0%	1.6%	0.8%	1.3%	0.3%	0.8%	1.0%	0.7%	0.6%	1.4%	0.7%	0.6%	1.3%	2.1%
E	3.4%	3.8%	3.7%	3.6%	3.0%	2.9%	4.9%	3.5%	5.0%	4.6%	4.4%	5.4%	4.0%	3.5%	4.9%	4.4%	8.8%	4.6%
F	0.7%	0.9%	0.8%	1.3%	0.6%	2.1%	1.1%	1.3%	1.7%	1.6%	1.6%	2.0%	1.2%	1.2%	1.6%	2.2%	3.0%	1.8%
G	11.6%	13.6%	10.7%	7.5%	9.4%	11.3%	12.6%	11.3%	17.8%	11.9%	11.5%	13.8%	11.8%	11.3%	13.8%	13.6%	8.1%	10.7%
H	1.0%	0.9%	1.1%	1.3%	0.9%	0.8%	1.1%	0.8%	1.0%	0.8%	0.8%	1.0%	0.9%	1.4%	1.0%	0.9%	2.0%	0.9%
I	0.7%	0.3%	0.5%	0.8%	0.4%	1.3%	1.4%	1.0%	0.3%	0.8%	1.0%	0.3%	1.2%	1.4%	0.3%	1.3%	0.3%	0.6%
J	0.5%	0.3%	0.3%	0.5%	0.4%	0.5%	0.3%	0.8%	0.7%	0.8%	1.0%	0.3%	0.3%	0.2%	0.7%	0.6%	0.7%	0.6%
K	7.4%	5.9%	6.7%	5.1%	5.8%	5.9%	6.0%	5.8%	7.0%	6.2%	6.3%	8.1%	6.3%	6.0%	8.2%	6.0%	4.7%	5.8%
L	9.1%	8.6%	5.6%	11.1%	9.6%	12.3%	6.8%	8.8%	4.7%	8.4%	7.3%	9.4%	8.9%	8.5%	9.5%	8.2%	5.7%	5.5%
M	7.1%	7.4%	7.7%	6.7%	6.2%	8.6%	9.3%	6.8%	8.7%	7.9%	8.9%	7.0%	6.9%	7.8%	6.9%	7.6%	7.1%	4.9%
N	0.7%	0.9%	0.8%	0.8%	0.6%		0.8%	0.8%		0.8%			0.9%	0.7%		0.9%		0.9%
O	0.2%	1.2%	0.3%	0.5%	0.9%	0.8%	0.3%	1.3%	0.7%	0.8%	1.3%		0.9%	0.9%		0.9%	0.7%	1.5%
P	3.7%	3.6%	3.7%	3.9%	2.8%	2.7%	4.9%	3.0%	5.4%	3.0%	2.9%	3.4%	3.2%	4.6%	3.3%	3.8%	5.1%	4.0%
Q	0.2%		0.3%	0.8%				0.3%	0.7%	0.8%	0.8%	0.7%	0.3%	0.5%	0.7%	0.6%	1.0%	0.9%
R	6.7%	7.1%	7.7%	5.9%	6.6%	5.4%	7.4%	7.8%	8.7%	7.3%	8.6%	7.0%	6.3%	6.9%	6.2%	7.6%	9.4%	8.9%
S	35.2%	33.7%	35.2%	37.5%	39.9%	33.2%	29.2%	35.5%	26.8%	31.7%	32.1%	28.2%	34.3%	32.7%	28.9%	30.6%	30.6%	34.3%
T	2.2%	2.4%	3.2%	2.6%	1.5%	1.9%	3.3%	2.3%	3.0%	3.5%	2.9%	2.7%	3.5%	2.1%	2.6%	2.5%	2.4%	3.1%
U	1.2%	1.2%	1.1%	1.5%	1.9%	0.3%	1.1%	1.0%		1.4%		0.3%	1.2%	0.9%	0.3%	1.3%	2.4%	1.8%
V	5.2%	5.6%	7.7%	6.2%	5.5%	6.4%	6.6%	4.8%	5.4%	5.4%	6.5%	7.4%	5.8%	5.8%	7.5%	4.7%	5.4%	5.5%

Figure S2

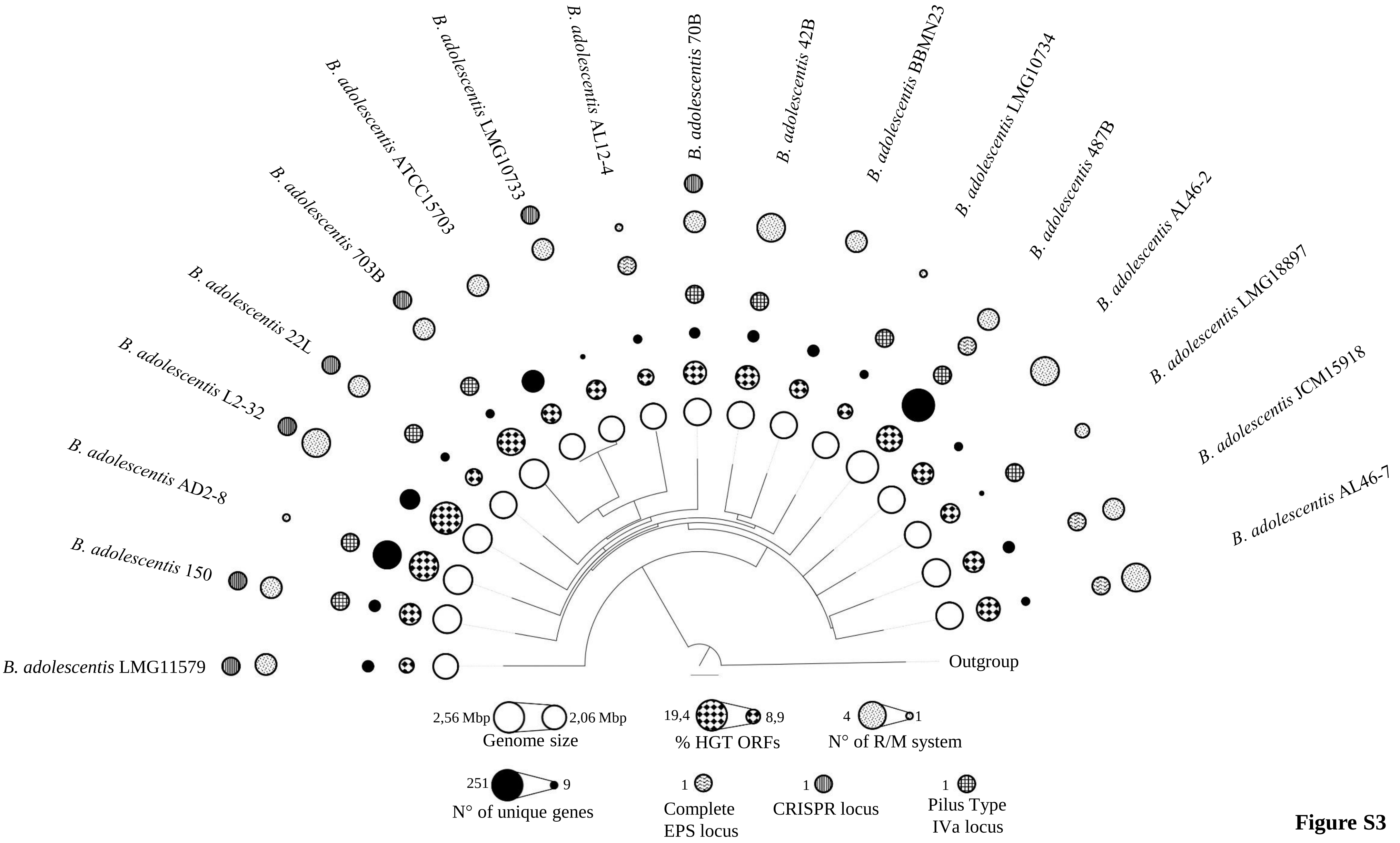
B. adolescentis LMG11579
B. adolescentis 150
B. adolescentis AD2-8
B. adolescentis L2-32
B. adolescentis 22L
B. adolescentis 703B
B. adolescentis ATCC15703
B. adolescentis LMG10733
B. adolescentis AL12-4
B. adolescentis 70B
B. adolescentis 42B
B. adolescentis BBMN23
B. adolescentis LMG10734
B. adolescentis 487B
B. adolescentis AL46-2
B. adolescentis LMG18897
B. adolescentis JCM15918
B. adolescentis AL46-7
Outgroup
2,56 Mbp
2,06 Mbp
Genome size
19,4
8,9
% HGT ORFs
4
1
N° of R/M system
251
9
N° of unique genes
1
Complete EPS locus
1
CRISPR locus
1
Pilus Type IVa locus

Figure S3

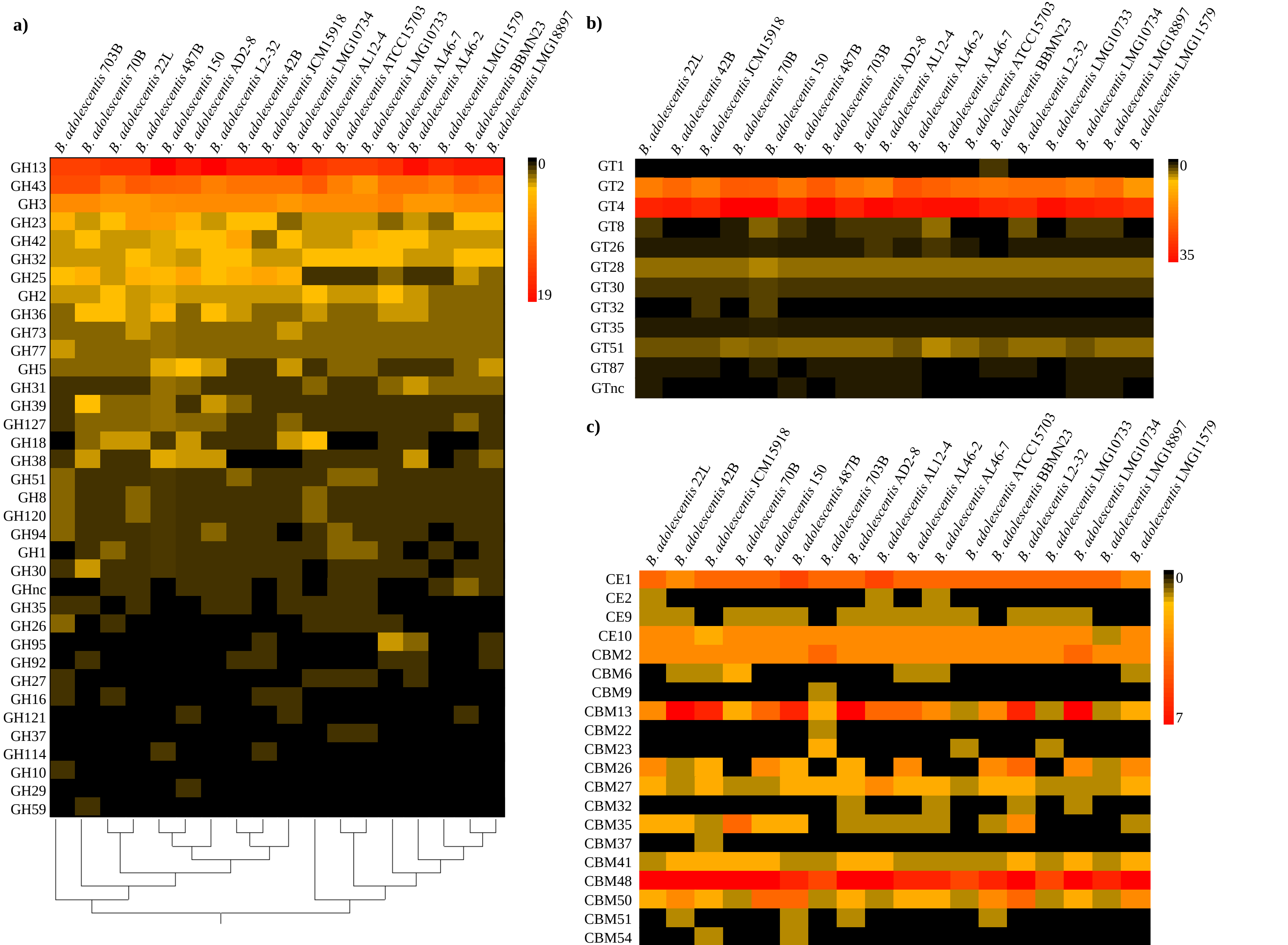
a)
B. adolescentis 703B
B. adolescentis 70B
B. adolescentis 22L
B. adolescentis 487B
B. adolescentis 150
B. adolescentis AD2-8
B. adolescentis L2-32
B. adolescentis 42B
B. adolescentis JCM15918
B. adolescentis LMG10734
B. adolescentis AL12-4
B. adolescentis ATCC15703
B. adolescentis LMG10733
B. adolescentis AL46-7
B. adolescentis AL46-2
B. adolescentis LMG11579
B. adolescentis BBMN23
B. adolescentis LMG18897
GH13
GH43
GH3
GH23
GH42
GH32
GH25
GH2
GH36
GH73
GH77
GH5
GH31
GH39
GH127
GH18
GH38
GH51
GH8
GH120
GH94
GH1
GH30
GHnc
GH35
GH26
GH95
GH92
GH27
GH16
GH121
GH37
GH114
GH10
GH29
GH59
0
19
b)
B. adolescentis 22L
B. adolescentis 42B
B. adolescentis JCM15918
B. adolescentis 70B
B. adolescentis 150
B. adolescentis 487B
B. adolescentis 703B
B. adolescentis AD2-8
B. adolescentis AL12-4
B. adolescentis AL46-2
B. adolescentis AL46-7
B. adolescentis ATCC15703
B. adolescentis BBMN23
B. adolescentis L2-32
B. adolescentis LMG10733
B. adolescentis LMG10734
B. adolescentis LMG18897
B. adolescentis LMG11579
GT1
GT2
GT4
GT8
GT26
GT28
GT30
GT32
GT35
GT51
GT87
GTnc
0
35
c)
B. adolescentis 22L
B. adolescentis 42B
B. adolescentis JCM15918
B. adolescentis 70B
B. adolescentis 150
B. adolescentis 487B
B. adolescentis 703B
B. adolescentis AD2-8
B. adolescentis AL12-4
B. adolescentis AL46-2
B. adolescentis AL46-7
B. adolescentis ATCC15703
B. adolescentis BBMN23
B. adolescentis L2-32
B. adolescentis LMG10733
B. adolescentis LMG10734
B. adolescentis LMG18897
B. adolescentis LMG11579
CE1
CE2
CE9
CE10
CBM2
CBM6
CBM9
CBM13
CBM22
CBM23
CBM26
CBM27
CBM32
CBM35
CBM37
CBM41
CBM48
CBM50
CBM51
CBM54
CBMnc
0
7

Figure S4

Table S1 Average nucleotide identity (ANI) alignment of *B. adolescentis* strains.

	B. adolescentis 487B	*B. adolescentis* AD2-8	*B. adolescentis* 22L	*B. adolescentis* JCM15918	*B. adolescentis* LMG18897	*B. adolescentis* BBMN23	*B. adolescentis* L2-32	*B. adolescentis* LMG11579	*B. adolescentis* ATCC15703	*B. adolescentis* 150	*B. adolescentis* 70B	*B. adolescentis* 703B	*B. adolescentis* LMG10734	*B. adolescentis* LMG10733	*B. adolescentis* AL12-4	*B. adolescentis* 42B	*B. adolescentis* AL46-2	*B. adolescentis* AL46-7
B. adolescentis 487B	---	98,47	98,68	98,25	98,52	98,4	98,27	97,83	98,17	98,52	98,45	97,91	98,59	98,17	98,31	98,44	98,29	98,18
B. adolescentis AD2-8	98,45	---	98,62	98,43	98,4	98,3	98,44	97,91	98,23	98,58	98,41	97,9	98,73	98,2	98,23	98,38	98,37	98,32
B. adolescentis 22L	98,65	98,59	---	98,41	98,5	98,45	98,47	97,95	98,29	98,55	98,43	98,27	98,51	98,28	98,36	98,48	98,39	98,33
B. adolescentis JCM15918	98,22	98,37	98,41	---	98,6	98,52	98,14	98,07	98,05	98,33	98,31	97,96	98,49	98,03	98,08	98,45	98,42	98,34
B. adolescentis LMG18897	98,5	98,34	98,51	98,61	---	98,53	98,51	98,04	98,04	98,58	98,32	97,97	98,5	98,03	98,11	98,45	98,63	98,5
B. adolescentis BBMN23	98,37	98,26	98,45	98,52	98,52	---	98,35	98,01	97,99	98,48	98,37	97,99	98,4	97,99	98,07	98,4	98,35	98,39
B. adolescentis L2-32	98,29	98,44	98,47	98,17	98,51	98,37	---	97,9	98,15	98,44	98,47	98,03	98,55	98,15	98,11	98,56	98,36	98,33
B. adolescentis LMG11579	97,83	97,85	97,95	98,08	98,04	98,02	97,89	---	97,91	97,96	97,89	97,89	97,97	97,88	97,84	97,77	98,01	98,05
B. adolescentis ATCC15703	98,13	98,17	98,29	98,05	98,04	97,99	98,13	97,91	---	98,12	98,23	98,67	98,28	99,93	98,48	97,99	97,92	98,03
B. adolescentis 150	98,53	98,56	98,57	98,36	98,58	98,49	98,45	97,96	98,14	---	98,42	98	98,53	98,11	98,15	98,36	98,49	98,42
B. adolescentis 70B	98,44	98,39	98,47	98,38	98,37	98,42	98,48	97,93	98,28	98,46	---	98,26	98,51	98,27	98,27	98,43	98,26	98,39
B. adolescentis 703B	97,93	97,89	98,3	97,98	98,01	98,02	98,01	97,92	98,7	97,99	98,24	---	98,16	98,68	98,42	98	97,86	97,91
B. adolescentis LMG10734	98,58	98,72	98,53	98,54	98,53	98,41	98,55	98	98,29	98,55	98,47	98,16	---	98,26	98,24	98,6	98,37	98,29
B. adolescentis LMG10733	98,13	98,16	98,3	98,05	98,03	97,99	98,14	97,89	99,93	98,11	98,23	98,67	98,27	---	98,47	97,98	97,91	98,01
B. adolescentis AL12-4	98,3	98,21	98,37	98,1	98,13	98,09	98,12	97,87	98,49	98,15	98,27	98,42	98,25	98,48	---	98,16	98,02	98,06
B. adolescentis 42B	98,44	98,42	98,53	98,5	98,45	98,42	98,56	97,8	98,06	98,39	98,45	98,04	98,61	98,03	98,15	---	98,36	98,29
B. adolescentis AL46-2	98,31	98,38	98,41	98,48	98,67	98,37	98,37	98,02	97,94	98,49	98,26	97,88	98,39	97,93	98,04	98,39	---	98,69
B. adolescentis AL46-7	98,18	98,33	98,34	98,37	98,51	98,41	98,33	98,05	98,04	98,42	98,37	97,91	98,3	98,01	98,05	98,28	98,68	---

Table S2 Percentage of genes putatively involved in HGT events.

	% ORFs
B. adolescentis 22L	20.93%
B. adolescentis 42B	8.68%
B. adolescentis 45T	24.27%
B. adolescentis 70B	41.99%
B. adolescentis 150	17.39%
B. adolescentis 487	33.72%
B. adolescentis 703B	25.51%
B. adolescentis AD2-8	41.99%
B. adolescentis AL12-4	22.50%
B. adolescentis AL46-2	16.09%
B. adolescentis AL46-7	11.11%
B. adolescentis ATCC15703	3.09%
B. adolescentis BBMN23	25.65%
B. adolescentis L2-32	33.60%
B. adolescentis LMG10733	4.17%
B. adolescentis LMG10734	15.65%
B. adolescentis LMG11579	25.87 %
B. adolescentis LMG18897	32.84%
